# 

**Published:** 1884-01-20

**Authors:** 


Abdominal Surgery .......... 460
Abortion.......... 41, 302, 318, 474
Abscesses.......... 108, 150, 274, 377
Achromatic microscope.......... 191
Acid, salicylic.......... 412
Acid, tartaric, in diphtheria.......... 314
Acne, rosea.......... 33
Adenitis, chronic.......... 356
Advice.......... 106, 299
After-pains.......... 110
Agaracin.......... 67
Ague.......... 466, 473
Air passages, foreign bodies in.......... 461
Air, watery vapor in the.......... 352
Alumnaria.......... 230
Alcoholism.......... 473
Aloes, mixture of.......... 190
Alum.......... 265
Amenities, ferryboat.......... 149
American medical association.......... 196, 239, 275
American editor's association.......... 77, 314
American public health association.......... 237
Ammonii, picras, in whooping cough.......... 473
Ammonia, valerianatis elixir.......... 314
Amyl, nitrate of.......... 30
Anæsthesia.......... 109, 114, 142, 248, 298, 427, 477
Anæsthetic, a new.......... 142, 427
Anaphrodisiac.......... 190
Aneurism.......... 1
Ani, pruritus.......... 235
Ano, fistula in.......... 230, 346
Anodyne.......... 76, 233
Anodyne, mixture, without opium.......... 238
Anthropophagy.......... 368
Anticholera infantis.......... 394
Antiferment.......... 300
Antipyrine.......... 412
Antiseptic gauze.......... 25
Antiseptics.......... 25, 67
Antiseptics in the puerpera.......... 25, 67
Antiquity of the trephine.......... 30
Antispasmodic mixture.......... 153
Antral abscess.......... 108
Aperient mixture.......... 194
Aphrodisiac.......... 76
Apomorphia.......... 219
Appetite of a bird.......... 190
Arsenic.......... 183, 209, 309
Arsenic, bromide.......... 183
Arsenicalis liquor, in nœvus.......... 309
Artificial food.......... 218
Ascarides.......... 235
Ascites.......... 374
Asclepias incarnata.......... 414
Aspirator.......... 425
Association, American medical.......... 196, 239, 275
Association, American medical editors.......... 237
Asthma.......... 73, 75, 150, 234, 236, 421, 433
Asthma mixture.......... 75
Astors, one of the.......... 337
Atlanta, mortality in.......... 397
Atlanta, too many physicians in.......... 475
Atmospheric glow.......... 112
Aural practice, electricity in.......... 253
Avena sativa.......... 99

B.

Bacillus.......... 78, 263, 350
Bacillus of cholera.......... 263
Bacillus of tubercle.......... 78, 350
Baldness.......... 27
Ballet, electric.......... 392
Balloon as a telegraphic medium.......... 231
Balsam of honey and tar (Hill's).......... 395
Baltimore medical association.......... 90
Bandage, a new form, for the eye.......... 148
Banyan tree.......... 301
Barlow, Dr. C., salicylic acid in rheumatism.......... 139
Basedow's disease.......... 252
Bathing.......... 105
Beaty, Dr. T. M., erysipelas.......... 130
Bed sores.......... 380
Bed-pan in child-bed.......... 187
Belladonna.......... 20, 69
Belladonna plaster, caution against.......... 69
Balladonna toxis, dose of, Dr. H. Gibbons.......... 20
Bellamy, Dr. W. C., on Gossydium.......... 283
Bemiss, death of Professor.......... 475
Benzoate of soda.......... 309
Bettman, Dr. B., celloidin.......... 375
Bichloride of mercury.......... 376
Biliary calculi, Dr. C. S. Webb.......... 93
Biliary colic.......... 110
Bilious dysentery.......... 234
Biography of Cotemporary Physicians of Texas.......... 475
Bird's appetite, a.......... 190
Birds, migration of, and cholera.......... 350
Bitters.......... 36, 73
Blackwood, Dr. R. D., electricity.......... 251
Bladder, distended, mistaken for a tumor, Dr. C. H. Leonard.......... 323
Bladder, irritable.......... 76
Bladder worm.......... 244
Bleeding, novel mode of.......... 150
Blindness from ophthalmia neonatorum.......... 348
Blistering, humane.......... 269
Board, National, of health.......... 262
Boarder, a.......... 149
Bodies for dissection.......... 308
Bodies, foreign, in air passages.......... 461
Bodies, foreign, in eye and lids, Dr. S. L. Frank 163
Boils.......... 225, 306, 472
Boils and carbuncles.......... 225
Boracic acid.......... 225
Borate of quinine.......... 412
Bowditch's formula.......... 26
Brain, abscess in the.......... 377
Brain, galvanization of the.......... 252
Brain substance, escape of.......... 390
Brandreth's pills.......... 35
Breathing deep.......... 383
Bromethyl.......... 459
Bromides.......... 268, 389
Bromides in diarrhœa of children.......... 268
Bromidia and morphia in delirium tremens.......... 382
Bromoform, a new anæsthetic.......... 142, 459
Bronchitis, Dr. Wm. H. Hamer.......... 418
Burn ointment, unrivalled.......... 353
Burns and scalds, Dr. G. O. Smith.......... 342
Bursting of coffin.......... 269

### C.

Cable, mending a submarine........ 191
Cadavers destroyed by sulphuric acid........ 111
Cadavers, preserving........ 29
Caffein........ 261
Calcium sulphide........135, 266
Calculation, a curious........ 391
Calculi........93, 188
Calculi, biliary, Dr. C. S. Webb........ 93
Calculus, renal........ 188
Caldwell, Dr. C. M., calcium sulphide........ 135
California laurel........ 178
Calomel........74, 260, 293
Calomel as a nervous sedative........ 260
Calomel as a rat poison........ 74
Calomel in true croup........ 293
Campbell, Dr........ 436
Cancer........ 476
Cancer hospital in New York........ 337
Cannabis indica........ 263
Cannabis, tannate of........ 412
Canned foods........ 221
Carbolic acid........25, 150, 192, 193, 241, 268
Carbolic acid and the Devil........ 192
Carbuncles and boils........ 225
Cardiac dropsy........ 344
Carotid artery, ligation of, Dr. Thad. Johnson 1
Cascara sagrada........ 63
Case (law) against Drs. Bomer & Keates........ 258
Case of John Jolly, Dr. A. W. Reese........ 8
Cass, Dr. Ed., conception without intromission........ 256
Castration of women........ 378
Cataract operation........ 298
Catarrh........356, 388
Caustic, Emarch's........ 393
Cerebro-spinal meningitis........105, 414
Cerium, valerionate of, in pregnancy........ 313
Celloidin, Dr. B. Bettmann........ 375
Chancres and old sores........ 181
Chapped hands and frosted feet........69, 114
Chicago medical society........398, 477
Chilblains........ 36
Child's origin, my........ 463
Childbed, bed-pan in........ 187
Children, artificial food for........ 218
Children, bringing up........ 312
Children, diarrhœa of........253, 268, 315
Children, how to give quinine to........ 75
Children, nocturnal terrors in, Dr. E. van Goidtsnoven........ 322
Children, summer complaints of........ 338
Chisolm, Dr., enlarged tonsils........ 227
Chloral, compound oleate of........ 348
Chloral in puerperal eclampsia........ 99
Chlorate of potassium, death from........ 348
Chlordes, Goodell's mixture of........ 114
Chloride of zinc in gonorrhœa........ 154
Chloroform........150, 265, 230, 300
Chloroform, albuminaria in........ 230
Chloroform as an antiferment........ 300
Chloroform narcosis........ 265
Chloroform, self-administration of........ 150
Cholagogue, Osgood's........ 473
Cholera........106, 219, 224, 263, 276, 336, 344, 350, 355, 364, 366, 367, 387, 388, 390, 398, 420, 432, 433, 455, 474
Cholera bacillus........ 263
Cholera and migration of birds........ 350
Cholera drops........ 355
Cholera, French theory on, Dr. M. Lereboulet. 364
Cholera germ........106, 420, 432
Cholera, German, commission........ 224
Cholera infantis, acute........ 394
Cholera mixtures........355, 393
Cholera preventives........ 390
Cholera, quassia as a cure for, Dr. H. B. Evans 367
Cholera, researches of Dr. Koch........ 455
Cholera report, Dr. Koch's........ 219
Chorea........ 252
Cider........ 384
Claricle, fracture of the........ 424
Clay, red, a remedy for sprains, Dr. G. G. Roy.. 442
Cleveland, Governor, and the Homœopaths... 397
Climate in Upper Georgia........ 226
Clinics in the Southern Medical College........ 436
Clock, a wonderful........ 431
Cloth, metallic........ 111
Coca........ 207
Cocaine, experience with hydrochlorate of...... 458
Cocoa........ 387
Cod liver oil........ 270
Codeia, syrup of........ 274
Coffee antidote to cholera........ 388
Coffee, syrup of........ 76
Coffin, bursting of a........ 269
Coincidence, singular........ 189
Cold, true nature of........ 66
Coleman, Dr. W. M., diphtheria........ 202
Colic, biliary........ 110
Collapse and sinking........ 343
Collodion........ 63
Color, red, for show globes........ 395
Colorado, coal mines of........ 72
Commencement exercises of Southern Medical College........ 115
Compass plant........ 419
Conception without intromission, Dr. Ed. Cass 239
Confidential communications........ 256
Congestive headache........ 195
Congress protecting physicians........ 239
Conium in malarial disease........ 388
Conjunctivitis, granular........ 30
Constipation........29, 245, 304
Constipation, emema in........ 304
Constipation habit, Dr. W. J. Fairfield........ 245
Consumption........310, 387
Consumption from street dust........ 310
Contagious disease, Tyndall's theory........ 380
Contrivance, a new........ 27
Convalescence, signs of........ 345
Convallaria majalis........ 218
Convulsions........180, 219
Convulsions, gelseminum in........ 180
Convulsions, infantile........219, 468
Copaiba, emulsion of........ 194
Copulation, excessive........ 426
Cord, spinal........ 209
Cornea, opacities of the........ 301
Cornea, painful ulcers of the........ 302
Corns, sure cure for........ 69
Corrosive sublimate........68, 209, 312
Corrosive sublimate in diphtheria........ 200
Corrosive sublimate in gonorrhœa........ 68
Cortelyofl, Dr. P. R., on Georgia climate........ 226
Cotoin........ 313
Cotton, absorbent, as a dressing........ 104
Cough........75, 274
Cough mixture........ 75
Coughing needless—useless........ 75
Couveuse, what is a........ 104
Criminal abortion........ 318
Crede's method of expression of placenta........ 465
Croton chloral........25, 33
Croton chloral in insomnia........ 25
Croton chloral in whooping cough........ 33
Croup, calomel in........ 293
Curiosities of the deep sea........ 315

### D.

Dandruff........ 35
Daniel, Dr. F. E., biography of Texas physicians........ 475
Dead, disposal of the, Dr. F. Gandrum........ 329
Death from chlorate of potassium........ 348
Deep sea, curiosities of the........ 351
Delirium tremens........64, 302, 382, 383
Dennett, Dr. H. E., dental organs........ 249
Dental organs, Dr. H. E. Dennett........ 240
Dental practice........ 392
Dentistry in the United States........ 312
Depilatories........ 35
Disinfectants........ 310
Detroit medical and library association........ 173

Dewar, Dr. John, ergot in whooping cough...... 176
Diabetes....135, 173, 183
Diabetes, a speedy fatal case of.... 173
Diabetes mellitus, Dr. C. M. Caldwell.... 135
Diagnostic sign of tuberculosis meningitis...... 161
Diarrhœa....234, 253, 309, 315, 434
Diarrhœa and dysentery.... 315
Diarrhœa and dysentery of children.... 315
Diarrhœa dysenteric.... 234
Diarrhœa in children....253, 315
Diarrhœa infantile.... 309
Diarrhœa, Squibb's, mixture.... 234
Difficulties, success under.... 343
Digitalis....490, 435
Diphtheria....44, 68, 173, 202, 209, 314, 342, 410
Disease, a new.... 390
Disease, germ of.... 216
Disease, malignant, Dr. H. M. Lawson.... 121
Diseases, contagious.... 380
Dislocations of the hip joint.... 141
Dislocations, reduction of H. M. L. Hutchinson.... 257
Dissecton.... 308
Diving bell, pressure in a.... 192
Dixon, Dr. Arch., abortion.... 41
Doctor, a liberal.... 197
Doctors and patients.... 239
Doctors, friendship of.... 38
Doctors in the West.... 349
Dogs and cats, scarlet fever in, Dr. J. C. Peters.. 170
Door paper.... 391
Drake, Dr. W. G., death of....358, 397
Dreams.... 272
Dressing, absorbent cotton as a.... 104
Dropsy....5, 26, 344, 458
Dropsy, cardiac.... 344
Dropsy, clinical remarks on, Dr. G. G. Roy...... 5
Dugas, death of Professor.... 438
Dugong.... 231
Dye, new hair.... 430
Dynamite in a boarding house.... 149
Dynamite, new process of making.... 152
Dysenteric diarrhœa.... 234
Dysentery....234, 306, 435
Dysmonorrhœa....273, 315, 384
Dyspepsia....95, 113, 114, 263, 474
Dyspepsia, chronic.... 263
Dyspepsia, flatulent.... 114
Dyspepsia, hepatic, Dr. T. H. Line.... 95
Dyspepsia, with constipation and piles.... 113
Dyspnœa.... 225

E.

Ear one foot in diameter.... 294
Earth, the, more rigid than steel.... 112
Eczema....35, 73, 422
Eczema, acute.... 73
Eczema of the scalp of infants.... 35
Electric ballet.... 392
Electric headlights.... 151
Electric power, a new.... 32
Electric treatment of perimetritis.... 469
Electricity in dental practice.... 392
Electricity in the slaughter house.... 152
Electricity, medical and surgical, Dr. R. D. Blackwood.... 251
Electricity, tanning by.... 471
Electro-generative torch or candle.... 151
Emmenagogue....395, 466
Enemas in constipation.... 304
Enteric fever.... 345
Epithelioma, Dr. H. M. Lawson.... 441
Ergot in dysmenorrhœa.... 384
Ergot in whooping cough, Dr. John Dewar..... 176
Ericsson's new solar motor.... 272
Erigeron Canadensis.... 350
Errors, surgical.... 222
Erysipelas....131, 241, 385
Erysipelas; Dr. J. McF. Gaston.... 241
Erysipelas in a child.... 385
Erpsipelas flegmonous, a case of, Dr. T. M. Beaty.... 130
Escharotics....309, 353
Esmarch's painless caustic.... 393
Ether douche, Dr. C. H. Hughes.... 23
Etherization by rectum.... 270
Ethyl, iodide of.... 113
Eucalyptus....30, 270
Evans, Dr. F. A., remittent fever.... 22
Evans, Dr. H. B., cholera.... 367
Eye, diseases of the, Dr. J. M. Roy.... 17
Eye and lids, foreign bodies in, Dr. S. L. Frank 163
Eye bandage, a new form of.... 148
Eye water....394, 472
Exophthalmic goitre.... 68
Extremities, numbness of upper.... 465

F.

Fahnestock's vermifuge.... 474
Fairfield, Dr. W. J., constipation.... 245
Febrifuge, salisylic acid and spirit of nitre, as a.... 466
Feeding infants too much.... 268
Feet, frosted....69, 469
Ferryboat amenities.... 149
Fever.... 429
Fever and kairin.... 420
Fever, intermittent....265, 267, 270, 423
Fever, typhoid.... 429
Fever, typho-malarial.... 307
Fevers, neutral treatment in, Dr. L. L. Holcombe.... 290
Fistula in ano.... 230
Flatulence....148, 347
Floors, polish for.... 312
Fœtus in extra uterine pregnancy.... 212
Follies, seven common surgical, Dr. J. B. Roberts.... 131
Food, artificial, for children.... 218
Foods, canned.... 221
Forehead, fracture of the.... 390
Formula for S. S. S.... 394
Fracture of the forehead.... 390
Fractures, treatment of, plaster of Paris.... 65
Frank, Dr. S. L, foreign bodies in eye and eyelids.... 163
French theories on cholera, Dr. M. Lereboulet 364
Friends.... 432
Friendship of doctors.... 38

G.

Galactagogue....252, 346
Galvanization of the brain.... 252
Gaston, Dr. J. McF., erysipelas.... 241
Gaston, Dr. J. McF., intestinal wounds.... 361
Gaston, Dr. J. McF., obstruction of ureter.... 447
Gaston, Professor....396, 431
Geigley, Dr. J. S., spermatorrhœa.... 166
Gelseminum in aftrr-pains.... 110
Gelseminum in convulsions.... 180
Georgia, Upper, climate of, Dr. P. R. Cortelyou 226
Germ of cholera.... 420
Germ of disease Dr. W. S. Newton.... 216
German cholera commission.... 224
Gibbons, sr., Dr. H., belladonna.... 20
Glass, a piece of, through the digestive tract... 467
Gleet treated by santonine.... 209
Globes, show.... 395
Globus histericus.... 390
Glue or paste for labels.... 36
Glycerine after bathing.... 105
Glycerine in febrile conditions.... 193
Glycerine in indigestion.... 380
Glycerite of tar....74, 383
Goldtsnoven, Dr. E. van....88, 161, 322
Goitre, exophthalmic.... 68
Goitre, extirpation of.... 266
Gold articles, tarnished.... 96
Gonorrhœa....28, 34, 68, 154, 472
Goodell's mixture.... 144
Goodwyn's syrup of hypophosphites.... 476
Gossypium (cotton plant), Dr. W. C. Bellamy... 283
Gout mixture, Laville's.... 34

Government harness dressing........................ 435
Gratitude to a physician........................ 190
Greely expedition........................ 369
Gross, Dr. S. D., death of........................ 196
Gross, Prof. S. W., on carbolic acid........................ 150
Gum, a new chewing........................ 195
Gundrum, Dr. F., disposal of the dead........................ 329

H.

Hæmorrhoids........................190, 193, 213
Hair dye, new........................ 430
Hair, effect of pilocarpine on the........................ 150
Hair tonic........................ 233
Hall's solution of strychnia........................ 354
Hamamelis Virginica........................ 427
Hamer, Dr. Wm. F., pneumonia, bronchitis, etc........................ 418
Hamilton, Dr. George, health of women........................ 210
Hamilton, Dr. S. L........................ 436
Hands, chapped, and frosted feet........................69, 469
Harvey's remains........................ 28
Hay fever........................ 143
Headache, congettive........................ 195
Headache, nervous........................ 473
Health, Illinois State board of........................ 337
Heart, irregular, Bowditch's formula........................ 26
Hemorrhoids........................ 462
Hernia, radical cure of........................ 103
Hernia, strangulated........................105, 213
Herr, Dr. F. C., Hæmorrhoids........................ 213
Hiccough........................ 148
Hill's balm of honey and tar,........................ 345
Hilarity, to produce........................ 153
Hip-joint, dislocations of........................ 141
Hippnrate of soda........................ 107
Hirsh, Dr. A. B., poisoning from Winslow's soothing syrup........................ 456
Hobbs, Dr. A. G., errors of refraction and accommodation........................ 82
Hodge, Dr. J. K., malarial hæmorrhagic fever.. 446
Holcombe, Dr. L. L., fevers........................ 290
Home journals........................ 437
Homœpaths and Governor Cleveland........................ 397
Homœopathy and New York medical society.. 437
Homœopathy in the United States........................ 101
Honey and tar, balsam of........................ 395
Hopkins, Dr. J. G........................ 436
Hostetter's bitters........................ 73
Hot water in inflamed mucous surfaces........................ 379
Hughes, Dr. C. H., ether douche for local pain 23
Hugo, Victor, on sewage irrigation........................ 271
Hutchinson, Dr. H. M. L., dislocations........................ 257
Hydragyrum, bichloridum, in dysentery........................ 306
Hydriodic acid, syrup of........................ 342
Hydrocele........................ 390

I.

Illinois State board of health........................ 337
Impotence, Dr. C. A. Bryce........................ 334
Incubation of infants........................ 181
Indigestion, glycerine in........................ 380
Indigestion, intestinal, Dr. L. Starr........................ 326
Indifference of Southern physicians........................ 437
Infant feeding........................ 268
Ingrowing toe-nail, Dr. R. C. Word........................ 13
Injections for piles, Dr. T. B. Washburn........................ 372
Insanity, bromides in........................ 389
Insomnia, croton chloral hydrate........................ 25
Intermittent fever........................265, 267, 270, 423
International medical congress........................106, 469
Intestinal obstruction by entero puncture........................ 141
Intromission, conception without, Dr. Ed. Cass........................ 256
Inunction, medicine by........................ 62
Iodide of potassium in typhoid fever........................ 24
Iodine in intermittent fever........................ 270
Iodine, tincture, in variola........................ 29
Iodoform in ophthalmia practice........................28, 110
Ipecacuanha in cholera........................ 23
Iron, tinclure, disguising the taste of........................ 195
Itch ointment........................ 75
Itch, soldiers'........................ 273
Ivy street hospital........................ 317

J.

Jequerinty, caution against........................ 221
Johnson, Dr. J. Thad........................357, 476
Johnson, Dr. J. Thad., ligation carotid artery.. 1
Jolly, case of John, Dr. A. W. Reese........................ 8
Journal, Our........................432, 475
Journals, Home........................ 437
Jurisprudence, medical, James A. Riley, esq... 370

K.

Kaoline pastes........................ 422
Keely motor........................ 470
Kitchen adornment, Oscar Wilde........................ 287
Koch, Dr. Robert, cholera researches........................ 155
Koch,s, Dr., cholera report........................ 219

L.

Labor, irregular contraction in........................ 174
Labor, to shorten, Dr. John Morris........................ 203
Laville's gout mixture........................ 34
Law case against Drs. Bowen & Keates........................ 258
Lawson, Dr. H. M., epithelioma........................ 441
Lawson, Dr. H. M., malignant disease........................121, 276
Lawson, Dr. H. M., orchitis........................ 95
Lead........................ 209
Lead paste........................ 422
Lemon, a decoction of, a cure for ague........................ 466
Leonard, Dr. C. H., distended bladder mistaken for a tumor........................ 323
Lereboulet, Dr. M., cholera........................ 364
Leucorrhœa, menstrual, Dr. S. C. Parsons........................ 201
Liberality of a Macon drug house........................ 476
Liaation, primitive, carotid artery, Dr. Thad. Johnson........................ 1
Light, effects of, on the eye........................ 110
Lightning stroke........................ 470
Line, Dr. T. H., hepatic dyspepsia........................ 108
Lidiment for rheumatism........................ 14
Liquor, hydrangyri bichloridi, in dysentery... 306
Liver, disease of........................ 374
Lobelia........................189, 414
Logan, Dr. John H........................ 436
Loganin........................ 312
London, mean temperature of, in January and July........................ 471
Longevity........................ 346
Loomis' tonic bitters........................ 314
Lumbago........................33, 149
Lung extirpation........................ 343
Lying-in room........................ 185

M.

Madness, frontiers of........................ 311

Malaria........................113, 144, 389, 426, 446

Malarial hæmorrhagic fever, Dr. J. K. Hodge.. 446

Male or female offspring at will........................ 385

Malignant disease, Dr. H. M. Lawson........................ 121

Malignant tumors........................ 223

Mammæ, inflamed........................ 177

Man vs. monkeys, dogs et al., Dr. van Goidtsnoven........................ 88

Mastitis........................ 134

Materia medica........................ 207

Measles, treatment of........................ 101

Medical association, American........................ 196

Medical association, Baltimore........................ 99

Medical association, Georgia........................77, 118, 156

Medical congress, International........................ 106

Medical education........................ 155

Medical literature in the South........................ 358

Medical progress........................ 37

Medical schools of Louisville........................ 155

Medical society of Chicago........................ 398

Medical society of West Virginia........................ 398

Medical students........................ 276

Medicine by inunction........................ 62

Medicines, administering........................ 186

Medicines, the, physicians use........................ 464

Medicines, to disguise the taste of........................ 94

Meningitis........................105, 161, 414

Menorrhagia........................ 389

Mercury, a new preparation of........................ 468

Metallic cloth........................ 111

Mercury....209, 376
Metric system.... 473
Microcephalic, oldest living.... 431
Micrococci.... 27
Microscope, achromalic.... 191
Migraine.... 100
Morphia and bromidia in delirium tremens... 382
Morphia habit and its treatment, by Dr. B. W. Richardson.... 108
Morphia, injection of, in infantile convulsions 468
Morphia, injection of, in strangulated hernia.. 105
Morphia with quinine.... 106
Morris, Dr. John, to shorten natural labor.... 203
Mortality in Atlanta.... 397
Mounteagle as a summer resort.... 358
Mucous surfaces, inflamed.... 379
Mudd, Dr., thyroid gland weighing 16½ ozs.... 332

N.

Nævus....146, 309
Narcosis, chloroform.... 265
Nasal hæmorrhage.... 304
Nasal polypi.... 182
Nashville Negro college.... 159
Nervous sedative, calomel.... 260
Neuralgia....69, 74
Neuralgic dysmemorrhœa.... 273
New Orleans, World's fair.... 316
Newton, Dr. W. S., germ of disease.... 216
New Yeur, the.... 37
New York medical society and homœopathy.. 437
New York post graduate medical school.... 77
Nicolson, Dr, William P., skull injury.... 281
Nickel, bromide of, in congestive headache.... 195
Night-sweats, agaracin in.... 67
Nose bleeding.... 474
Nose, plugging the.... 304
Number seven.... 381
Numbness of upper extremities.... 465
Nurses' sore mouth.... 306
Nux vomica as a galactagogue.... 346

O.

Obstetrical problems.... 285
Obstruction, intestinal.... 141
Offspring, factors deciding the sex of the.... 138
Offspring, male or female, at will.... 385
Ointment for itch.... 75
Oleate of chloral, compound.... 348
Oleic acid.... 113
Opacities of the cornea.... 301
Ophthalmia neonatorum....348, 421
Ophthalmia, yellow oxide of mercury, in.... 69
Ophthalmic practice, iodoform in.... 28
Ophthalmic therapeutics.... 110
Opium, alkaloids.... 208
Opium poisoning, nitrate of amyl.... 30
Opium, substitue for.... 270
Optic nerve, injections into the.... 29
Orchitis, Dr. H. M. Lawson.... 95

P.

Packard, Dr. John H., a case of imperforate rectum.... 452
Paraffine paper.... 311
Paraldehyde as a hypnotic.... 307
Paralysis, Dr. Philip Zenner.... 57
Parcels, Dr. W. H., puerperal eclampsia.... 288
Parham, Dr. E. H. M., bladder worms.... 244
Parker, Dr. William, death of.... 196
Parsons, Dr. S. C., menstrual leucorrhœa.... 201
Parvin, Dr. T. H., diarrhœa in children.... 253
Paste or glue for paper labels.... 36
Pasteur's laboratory.... 231
Pathological specimen, Dr. Mudd.... 332
Patients, a new contrivance for raising.... 27
Pericardiac and cardiac wounds.... 224
Perimetritis.... 469
Pernicioas intermittent fever.... 423
Peters, Dr. J. C., scarlet fever in dogs and cats.. 170
Phlebitis.... 384
Phlegmonous inflammation.... 213
Phthisis....33, 185, 356
Physicians, the, and the State.... 78
Physicians, Congress protecting.... 239
Physicians, Southern.... 427
Phytolacca decandra.... 117
Piles....14, 73, 262, 265, 372, 394, 466
Pilocarpine, effect of, on the hair.... 150
Pilocarpine in ascites.... 374
Pitting in small-pox.... 235
Plrcenta, expression of the, Crede's method.... 465
Placenta prævia....188, 261
Placenta, removal of.... 175
Placenta retained.... 259
Plaster of Paris, fracture dressed by.... 65
Plastic mass.... 32
Pleuretic effusion.... 303
Pleurisy, Dr. William F. Hamer.... 418
Pneumonia, Dr. Hamer.... 303
Poisoning by opium, nitrate of amyl.... 30
Poisoning by rattlesnake.... 147
Poisoning from Winslow's soothing syrup, Dr, A. B. Hirsch.... 456
Poisoning, instant remedy for.... 345
Poisoning, malarial.... 113
Potassium, permanganate, in rattlesnake poisoning.... 147
Powell, Dr. R. S.... 48
Pregnancy....212, 331, 386, 415
Progress, recent, in therapeutics.... 207
Pruritus....99, 235, 345, 475
Puerpera, antiseptics in the.... 67
Puerperal eclampsia....99, 288

Q.

Quassia in cholera.... 367
Quebracho in dyspnœa.... 225
Quinine....60, 75, 412, 434
Quinine, borate of.... 412
Quinine; how to give it to children.... 75
Quinine, oxytoxic action of.... 60

R.

Rat poison, calomel as a.... 74
Rattlesnake poisoning.... 147
Ray, Dr. J. Morrison, diseases of the eye.... 17
Recovery, remarkable.... 190
Rectum, etherization by.... 270
Rectum, imperforate, Dr. J. H. Packard.... 452
Reduction of dislocation, Dr. H, M. L. Hutchson.... 257
Reese, Dr. A. W., case of John Jolly.... 8
Remarks of Professor Gaston, Southern Medical College.... 401
Remittent fever, Dr. F. A. Evans.... 22
Renal calculus.... 188
Retrospect of medical progress.... 37
Reward offered, yellow fever.... 137
Rheumatism....18, 36, 76, 139, 224, 433
Richardson, Dr. B. W., on morphia habit.... 108
Riley, esq., James A., medical jurisprudence... 370
Ringworm.... 353
Roberts, Dr. John B., surgical follies.... 131
Roy, Dr. G. G., clinical remarks on dropsy.... 5
Roy, Dr. G G., red clay as a eemedy for sprains 444
Rupprecht on boils and carbuncles.... 225

S.

Sadberry spleen mixture.... 373
Salicylate of sordium....30, 76
Salicylic acid....30, 105, 139, 266, 414, 466
Salt.... 351
Sanguis bovinus exsiccatus, Dr. F. E. Stewart.. 97
Santonin....186, 209
Scabies.... 266
Scalp, dry seborrhœa of the.... 195
Scalp of infants, eczema of.... 35
Scarlatina.... 194
Scarlet fever in dogs and cats, Dr. J. C. Peters.. 170
Sciatica.... 340
Scrotal pruritus.... 235
Seborrhœa, dry, of the scalp.... 195
Secundines, immediate removal of, Dr. Arch. Dixon.... 41
Sedative, avena sativa, as a.... 99
Selenetropism.... 432
Sewage irrigation.... 271

Sewer arrangement, hygienic .... 431
Sewerage, Waring's system.... 278
Sex of offspring, factors deciding the.... 138
Sexual organs, weakness of.... 274
Sexual powers, drugs influencing.... 267
Sierra salviæ in remittent fever, Dr. F. A. Evans.... 22
Sight, defective, Dr. A. G. Hobbs.... 82
Skin diseases ....301, 422
Skull injury, Dr. William Perrin Nicolson.... 281
Skull, trephining.... 222
Skunk, the poison of the.... 305
Small-pox, pitting in.... 235
Smith, Dr. G. O., burns and scalds.... 342
Snake bite ....29, 35
Soda, benzoate of.... 309
Soda, hippurate of.... 107
Sodium bromide vs. potassium bromide .... 179
Solar motor, Ericssou's.... 272
Soldier's itch.... 273
Song of the tubercle bacillus.... 350
Sorghum in typho-malarial fever.... 307
South, medical literature in the.... 358
Southern Medical College....77, 115, 276, 357, 396, 436
Southern physicians .... 437
Specimen, pathological, Dr. Mudd.... 332
Spermatorrhœa ....166, 195, 334
Spinal cord.... 209
Spirit of nitre as a febrifuge.... 466
Spleen....233, 273, 295
Sputa of phthisical patients.... 185
Squibb's diarrhœa mixture.... 434
S. S. S., formula for.... 394
St. Gothard tunnel.... 72
St. Peter's reply to " My child's origin ".... 463
Stains, rust, on cotton and linen.... 435
Stammering, a cure for.... 140
Starch, iodide of, in intermittent fever.... 468
Starch pastes.... 423
Starr, Dr. L., intestinal indigestion.... 326
Steam heater, a pocket.... 151
Stewart, Dr. F. E., sanguis bovinus exsiccatus.. 97
Stomach, ulcer of the.... 463
Strychnia....302, 354
Stye....304, 354
Styptics.... 222
Styrian steel .... 31
Success under difficulties.... 343
Succinate of iron.... 110
Sugar from rags.... 31
Suicide .... 436
Sulphuric acid, cadavers destroyed by.... 111
Sulphuric ether.... 149
Summer complaint in children.... 338
Suppositories for piles.... 73
Surgeons, European vs. American.... 61
Surgical dressing, cod liver oil, as a.... 270
Surgical errors .... 242
Surgical follies.... 131
Sweating in phthisis.... 32
Syphillis.... 36
Syphillitic test.... 230
Syrup of coffee.... 76

T.

Tœnifuge.... 113
Tannate of cannabin.... 412
Tannin, a specific for carbuncles.... 468
Tanning by electricity.... 471
Tar, glycerate of....74, 383
Tartaric acid in diphtheria.... 314
Taste of medicine, to disguise the.... 94
Telegraph medium, balloon as a.... 231
Terra cotta lumber .... 31
Terrors, nocturnal, in children, Dr. E. van Goidtsnoven .... 322
Testimony, expert.... 436
Tetanus....69, 223, 424
Theine.... 469
Therapeutics and materia medica.... 207
Thermo-cautery in anal fistula.... 346
Thielemann's cholera mixture.... 393
Thompson's eye water .... 394
Tic douloureux .... 266
Toe nail....13, 390
Tolu, rock and rye.... 194
Tonga.... 180
Tongue in health and disease.... 141
Tonic bitters....36, 314
Tonsilitis....30, 34
Tonsil, enlarged....227, 473
Toothache.... 219
Tooth drawing.... 70
Tracheotomy.... 340
Traumaticin in diseases of the skin.... 301
Trephine, antiquity of the .... 30
Trephining the skull, danger of.... 222
Trinotine.... 183
Truth and love.... 298
Tubercle, bacillus of.... 78
Tuberculous material.... 212
Tumors, malignant.... 223
Turpentine as a prophylactic.... 221
Tyndall's theory of contagious diseases.... 380
Typhoid ; how it spreads.... 145
Typhoid fever....24, 75, 153, 429
Typho-malarial fever.... 307

U.

Ulcer of the stomach.... 463
Ulcers of the cornea.... 302
Ulcers, venereal.... 30
Upshur, Dr. John M., vomiting in pregnancy.. 415
Ureter, obstruction of, Dr. J. McF. Gaston.... 447
Urticaria.... 469
Uterine disease.... 305

V.

Vaccine virus.... 377
Valerianate of cerium.... 331
Vance, Dr. A. Morgan, anæsthesia.... 248
Vanilism.... 310
Vapor, watery, in the air.... 352
Variola.... 29
Varicose vein.... 269
Vaseline cerate.... 146
Veatch, Dr. William H., piles.... 14
Venereal sores and ulcers.... 30
Veratrum, substitute for .... 462
Vesication in diphtheria.... 68
Vermifuge, Fahnsiock's.... 474
Vomiting in pregnancy....331, 386, 415

W.

Walnut hair dye.... 314
Waring's system of sewerage.... 278
Warts....60, 308
Washburn, Dr. G. B., piles.... 372
Water cure in diphtheria.... 342
Watery vapor in the air.... 352
Webb, Dr. C. S., biliary calculi.... 93
West, doctors in the.... 349
West Virginia, medical society of.... 398
Whale, vegetarian.... 231
Whooping cough.... 33, 176, 473
Wilde, Oscar, on kitchen decoration.... 287
Winslow's soothing syrup, a case of poisoning from.... 456
Wire, silver, sutures, Dr. J. McF. Gaston.... 361
Witch hazel in menorrhagia.... 389
Witness, Medical, advice to the.... 299
Woman, castration of.... 378
Woman, health of, Dr. George Hamilton.... 210
Word, Dr. R. C., ingrowing toe-nail.... 13
World's Fair at New Orleans.... 316
Worms, bladder, Dr. E. H. M. Parham.... 244
Wounds of the intestines, Dr. J. McF. Gaston.. 361
Wounds, recent method of treating.... 23
Wounds, pericardiac and cardiac .... 224
Writing, easy.... 72

Y.

Yandell, Dr. L. P., death of.... 189
Yellow fever....237, 345

Z.

Zenner, Dr. Philip, paralysis .... 57

Zinc, chloride, in gonorrhœa .... 154

### *RECEIPTED.*

1883—Drs. T J Chailton, A R Oglesby, T S Fox. W N Ames, to July; A B McWhorter, G W Bowling, L T Boatright, Wm Johnson, L M Wood, Jeff Wilcox, J C Terrell, to July; J B Bailey, C Hamilton, to July.

1884.—Drs. W S Glass, J W Pettis, W B Sanders, A M Jamerson, W A Shands, N Y Acad, of Med; T S Parrham, D B Hamilton, W J McAlpine, A T Park, W T Kendall, John W Lee, W B King, R F Mathews, T P Olliver, J R Reeves, T J Brasher, J R Wilson, W H Boykin, Jno G Steed, J M Stansill, Nathan King, J E Frippe, W H Stewart, Z J Scott, R H Hargrove, A B Robinson, C P Sanders, J C Boyce, Tho H Sanders, H J Sanders, M W Speer, J M Underwood, Jno S Caruthers, to July '85; E S Foster, to July '85; B Chambliss, '82.

